# Targeted therapy for multiple gene mutations in multiple metastases of advanced gastric cancer: a case report

**DOI:** 10.3389/fonc.2023.1257011

**Published:** 2023-12-15

**Authors:** Xin Zhang, Xinran Zhang, Dandan Geng, Chenguang Zhao, Yingnan Wang, Yao Fan, Shasha Gao, Jinmei Wei, Fengbin Zhang

**Affiliations:** ^1^ Department of Gastroenterology, Fourth Hospital of Hebei Medical University, Shijiazhuang, Hebei, China; ^2^ Department of Neurology, The People’s Hospital of Hebei Province, Shijiazhuang, Hebei, China; ^3^ Department of Internal Medicine, Baoding Orthopedic Hospital/People’s Hospital of Lianchi District, Baoding, Hebei, China

**Keywords:** gastric adenocarcinoma, MET amplification, HER-2 amplification, targeted therapy, heterogeneity

## Abstract

In China, gastric cancer is the second most common cause of cancer-related death, after lung cancer. At present, the morbidity and mortality rates of gastric cancer are increasing, and targeted therapy for gastric cancer has become a research hotspot. Herein, we report a patient with multiple metastases from advanced gastric cancer. After identifying *MET* gene amplification, initial treatment induced regression of the tumor. However, in later stages, due to the overexpression or mutation of *HER-2*, *KRAS*, *TP53*, and other genes, the targeted drug therapy became ineffective, and the disease progressed rapidly, leading to the death of the patient.

## Introduction

With changing dietary and living habits, the incidence of gastric cancer has been increasing yearly. Gastric cancer is the fifth most common malignancy worldwide (5.7%) and the third most common reason for cancer-related mortality (8.2%) (1). In China, gastric cancer is the second most common cause of cancer-related death, after lung cancer (1). The symptoms of gastric cancer are often non-specific; therefore, most patients present in the advanced stage of disease by the time they present to a doctor. Traditional palliative chemotherapy has shown limited efficacy for advanced gastric cancer whereas for gastric cancer, targeted therapy has become a research hotspot. However, due to the spatial and temporal heterogeneity of gastric cancer, the selection of drug for use as targeted therapy is difficult.

## Case description

**Figure 1:**
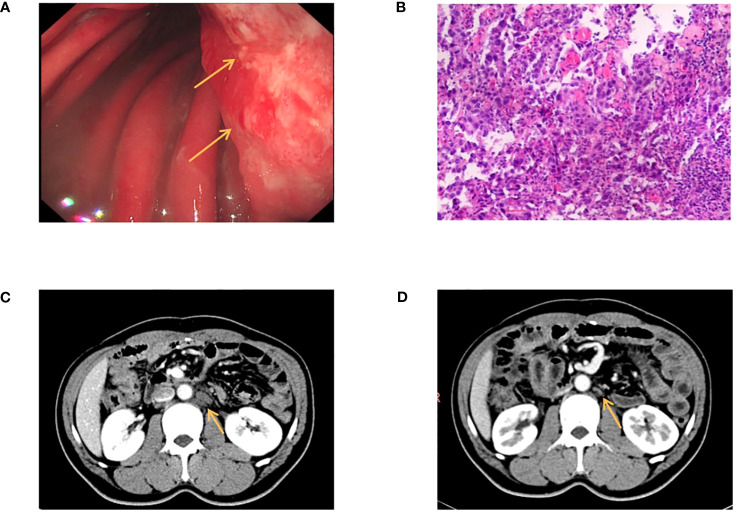
**(A)** Gastroscopy revealed ulcer-like lesions in the small curvature of cardia and gastric body. **(B)** Pathological examination of a biopsy specimen found adenocarcinoma. **(C)** Enhanced CT showed that the abdominal and retroperitoneal lymph nodes had increased and enlarged. **(D)** Enhanced CT after MET targeted therapy showed that the abdominal and retroperitoneal lymph nodes had decreased than anterior. The arrow in **(A)** indicate the locations of ulcer-like lesions in the small curvature of cardia and gastric body. The arrow **(C, D)** indicate the locations of enlarged retroperitoneal lymph nodes.

**Figure 2:**
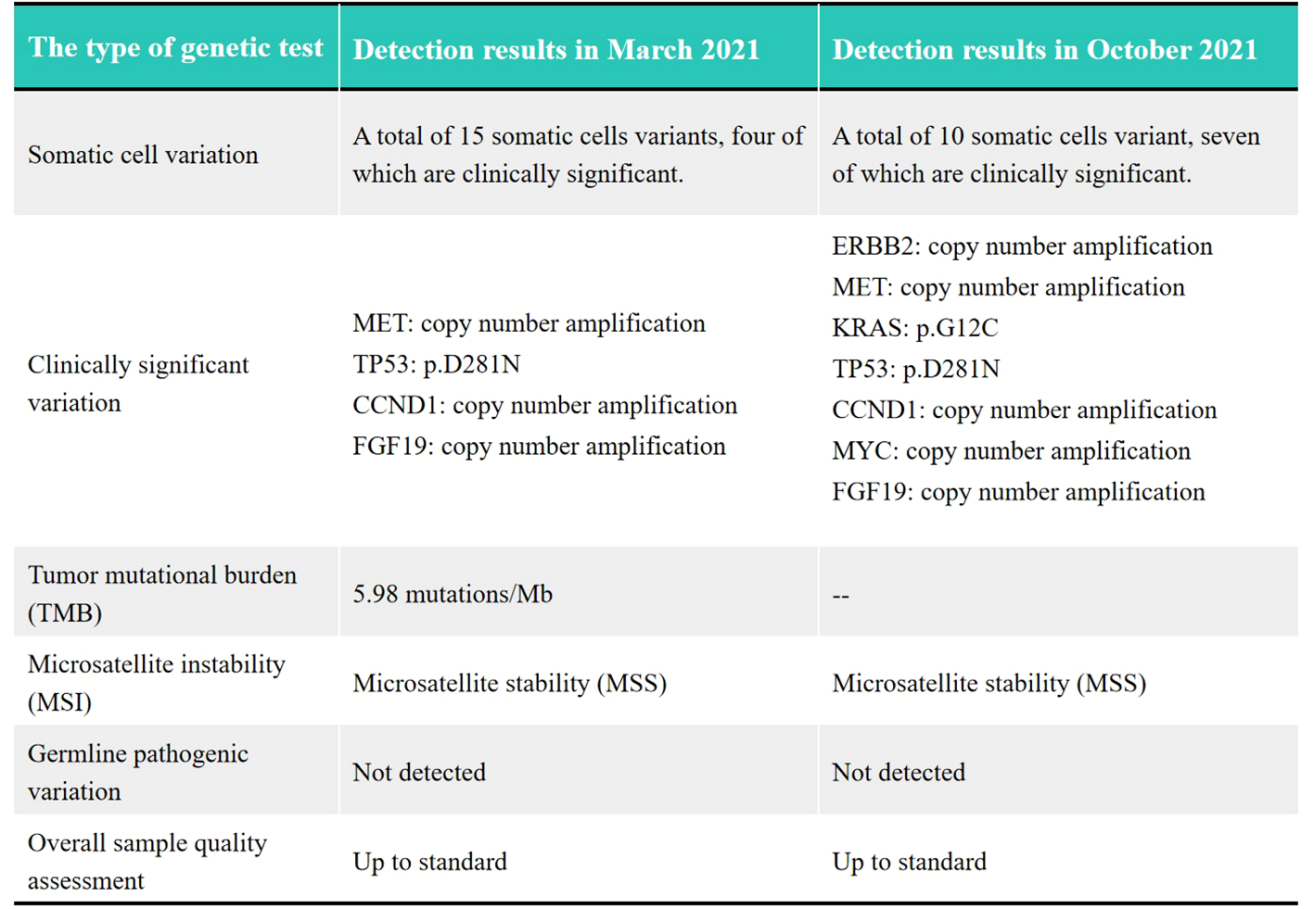
Comparison of genetic tests between March 2021 and October 2021. The symbol “–” means the TMB detection wasn’t performed in October 2021.

**Figure 3:**
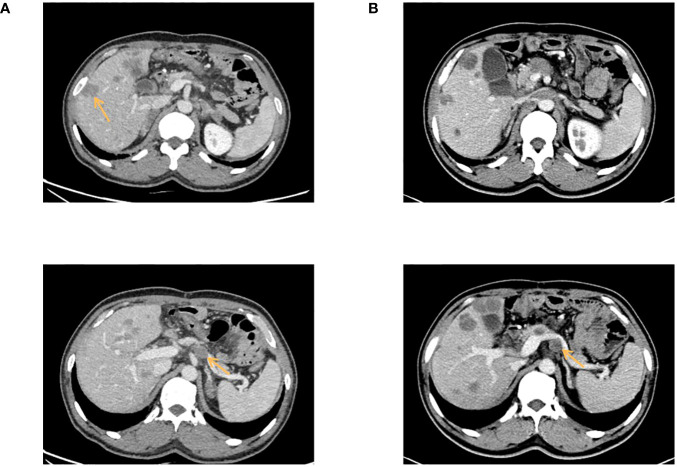
**(A)** Enhanced CT showed tha the liver had new metastasis. **(B)** Enhanced CT showed the maximum short diameter of abdominal and retroperitoneal lymph nodes was increased, and the maximum long diameter of intrahepatic metastasis was larged. The arrows indicate the locations of new metastases in the liver and the locations of enlarged retroperitoneal lymph nodes.

**Figure 4:**
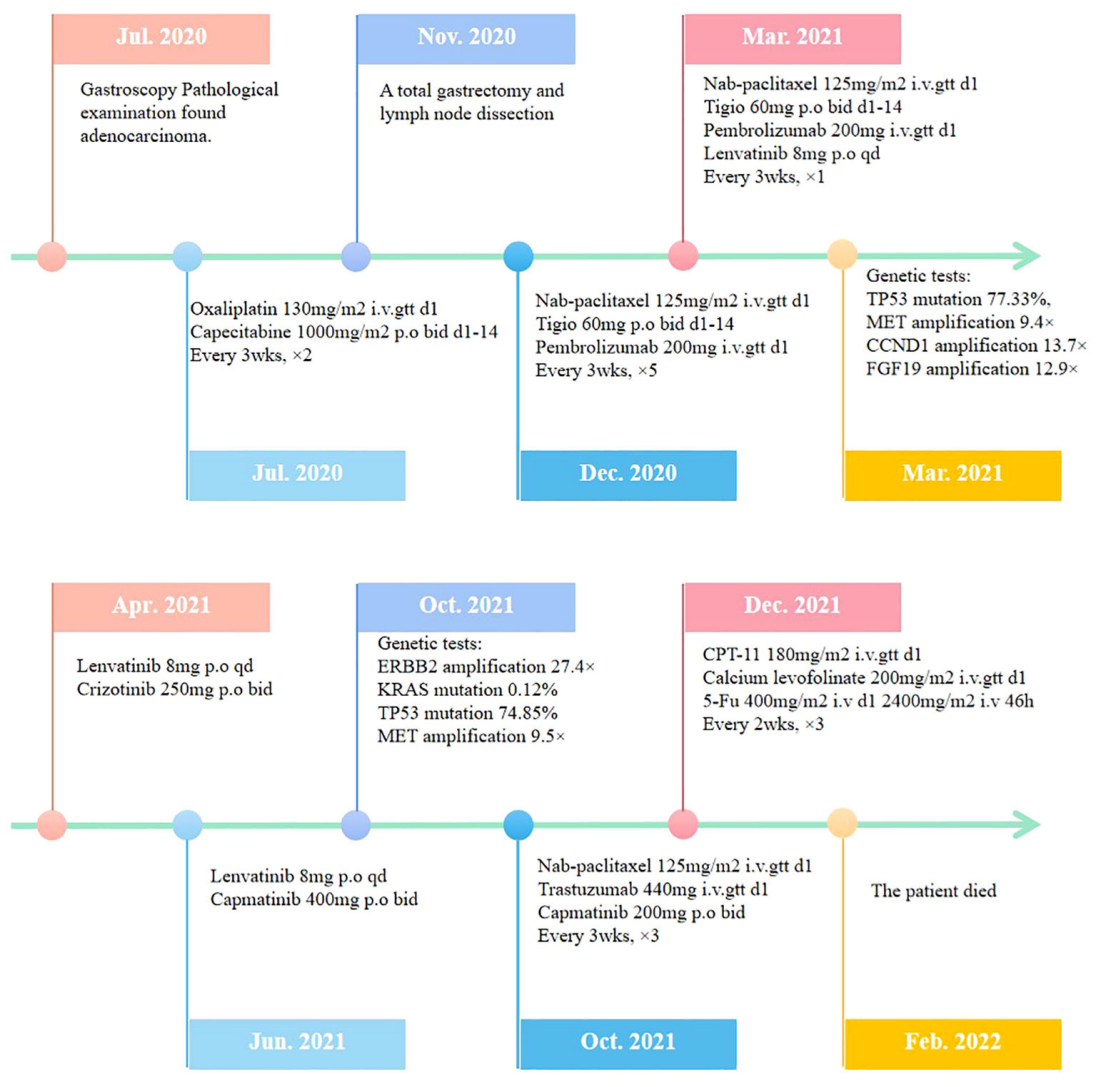
Timeline for diagnosis and treatment form July 2020 to Feb 2022.

The patient was a 41-year-old male with no family history of cancer, but had a history of excessive alcohol intake and had smoked for longer than ten years. He was admitted in July 2020 with a two-week history of “choking feeling following eating.” Gastroscopy revealed ulcer-like lesions in the small curvature of the cardia and gastric body (**Figure 1A**). Pathological examination of a biopsy specimen identified adenocarcinoma (**Figure 1B**). Enhanced computed tomography (CT) suggested that the lesion was Borrmann II localized ulcer type and the maximum thickness was 3.22 cm. Laparoscopic exploration revealed gastric adenocarcinoma stage III C (T4aN3M0). Two cycles of XELOX (oxaliplatin 150 mg d1+capecitabine 1.5 mg 2/d d1-14) was given between July 2020 and August 2020, combined with apatinib targeted therapy. Apatinib is an anti-vascular targeted drug, which was first developed, tested, and approved in China, its global efficacy and safety data are still being collected (2). In October 2020, positron emission tomography - CT (PET/CT) examination showed multiple lymph node metastasis in the para-abdominal aorta and left clavicular region. Total gastrectomy and lymph node dissection were performed in November 2020. Postoperative pathology revealed the presence of ypT0N1. Postoperative immunohistochemistry showed HER-2 (1+), MLH1 (+), MSH2 (+), PSM2 (+), MSH6 (+), EBER (-), and PD-L1 (+) CPS3. The Lauren classification indicated intestinal-type gastric cancer (IGC). One week postoperatively, enhanced CT revealed newly enlarged retroperitoneal lymph nodes (the shortest diameter of the largest lymph node was about 1.3 cm), and recrudescence was considered. After operation, AS combined with K drug (nab-paclitaxel 0.3 g d1+Tigio 60 mg 2/d d1-14+pembrolizumab 200 mg d1 q3w) regimen was used in five cycles. Evaluation of the target lesion, according to the RECIST 1.1 scoring standard, revealed enlarged stable disease (SD) in the fourth cycle (the abdominal cavity and retroperitoneal lymph nodes were increased and the largest lymph node short diameter was about 1.5 cm). To further strengthen the treatment effect, lenvatinib was added in the sixth cycle of treatment. Enhanced CT showed that the abdominal and retroperitoneal lymph nodes had increased and enlarged (the shortest diameter of the largest lymph node was about 1.6 cm) (**Figure 1C**). Genetic tests performed in March 2021 revealed *TP53* mutation (77.33%), mesenchymal-epithelial transition factor proto-oncogene (*MET*) amplification (9.4x), *CCND1* amplification (13.7x), and *FGF19* amplification (12.9x) (**Figure 2**). Treatment was changed to lenvatinib 8 mg 1/d+crizotinib 250 mg 2/d. After 50 days of treatment, CA19-9 levels decreased from 427 U/mL to 17.13 U/mL, and CA72-4 reduced from ≥ 300 U/mL to 16.16 U/mL. Enhanced CT after *MET* targeted therapy showed that the abdominal and retroperitoneal lymph nodes had decreased than previously (**Figure 1D**). Because the patient had a pulsatile headache, cranial magnetic resonance imaging (MRI) performed in June 2021 showed metastatic tumor (the cerebellar hemisphere, right occipital lobe, right centrum semiovale, and bilateral parietal lobe had multiple lesions, with a maximum diameter of 3.1 cm), which was evaluated as progressive disease (PD). Crizotinib was replaced with capmatinib, which crosses the blood-brain barrier. Head MRI after 2 months showed that the intracranial metastatic tumor was smaller than that seen in the previous film (cerebellar hemisphere, right occipital lobe, right centrum semiovale, and bilateral parietal lobe, with a maximum diameter of 2.3 cm). The lesion was evaluated as SD. After 2 months of continued oral capmatinib, CT revealed that the lesion was PD (the liver had new metastasis with a maximum diameter of 2.6 cm while the shortest diameter of the largest lymph node was 1.4 cm) (**Figure 3A**). No significant changes were observed in the head MRI results. Genetic analysis performed in October 2021 revealed that *ERBB2* copy number was amplified (27.4x), and there was *KRAS* mutation (0.12%), *TP53* mutation (74.85%), *MET* amplification (9.5x), *MYC* amplification (4.1x), *CCND1* amplification (14.5x), and *FGF19* amplification (14.3x) (**Figure 2**). Trastuzumab was added to the next line of treatment. The nab-paclitaxel+trastuzumab+capmatinib regimen was administered in three cycles, between October 2021 and November 2021. After one cycle, the levels of CA19-9 decreased from 2000 U/mL to 108.6 U/mL, CA72-4 showed no obvious changes, while CEA decreased from 12.51 ng/mL to 1.87 ng/mL. After two cycles, the lesion was evaluated as PD (CT showed new metastases with the shortest diameter in the mediastinal lymph nodes of 1.7 cm and in the abdominal and retroperitoneal lymph nodes of 2.4 cm, whereas, the longest diameter in the intrahepatic metastasis was 3.4 cm) (**Figure 3B**). MRI showed new metastasis in the thoraco-lumbar spine. Head MRI showed that the longest diameter in the bilateral cerebellar hemisphere and right parietal lobe lesions was 1.7 cm). However, the general condition of the patient was poor, with multiple metastases, multiple organ circulatory failure, disseminated intravascular coagulation (laboratory test results revealed fibrinogen of 0.88 g/L, platelets of 48,000/mL, prothrombin time of 22.1s, and D-dimer of 37 mg/L, with the indicators continuing to show deterioration), and failure to tolerate continued chemotherapy and targeted therapy. The patient died in February 2022 (**Figure 4**), with an overall survival of 19 months.

## Discussion

The overall prognosis of advanced gastric cancer is poor. When the initial standard treatment effect is poor, stratified precision treatment becomes popular. In gastric cancer, the most common aberrant genes include *TP53*, *PIK3CA*, *ERBB2*, *ERBB3*, *ARID1A*, and *KRAS*, and less commonly *MET*, *FGFR1*, *MYC*, and *CDH1* (3).

The *MET* is located on the long arm of human chromosome 7 and encodes a c-MET protein with tyrosine kinase activity. Hepatocyte growth factor (HGF) is the only ligand of c-MET (4). After HGF binds to c-MET, receptor homodimerization, autophosphorylation of carboxyl-terminal tyrosine residues, and activation of mitogen-activated protein kinase (MAPK), phosphatidylinositol 3 kinase (PI3K), and Rac1-Cdc42 signals occur (5), activating the downstream PI3K/AKT/mTOR and RAS/RAF/MEK/ERK pathways. *MET* abnormalities mainly include skip mutation of the MET14 exon, amplification of the *MET* gene, and abnormal expression of the c-MET protein (6). Among these, the overexpression of c-MET protein is the most common (50-65%) (7), followed by *MET* gene amplification; however, *MET* gene mutations rarely occur (8). *MET* gene amplification is usually mutually exclusive to other abnormally changed genes, and less likely to co-amplify (3.4-7%). *MET* amplification is usually found in IGC (9, 10). Crizotinib acts on the MET/ALK/ROS signaling pathway and is currently the most commonly used tyrosine kinase inhibitor (TKI). However, for gastric cancer with c-MET overexpression or mutation, the therapeutic effect still needs to be confirmed. In one study, tissues were collected from 262 patients with advanced gastric cancer, and cancer cells were treated with crizotinib. The findings showed that cells with a c-MET mutation and overexpression of MET protein were not sensitive to crizotinib. However, cells with amplified *MET* were sensitive to crizotinib (11). The patient we report also had C-met amplification and benefited from the treatment. This further demonstrates the efficacy of crizotinib in patients with *MET* gene amplification. Unlike crizotinib, capmatinib can cross the blood-brain barrier (12), making it the best choice for gastric cancer with *MET* amplification and brain metastasis.

In the late stage of disease progression, the *HER-2* (*ERBB2*) gene was amplified, and trastuzumab was specifically administered. *HER-2* activates and mediates the activation of downstream signaling pathways through the formation of heterodimers and autophosphorylation of tyrosine kinases (13). It is primarily involved in the Ras/MAPK/ERK and PI3K/Akt/mTOR pathways. When *HER-2* is abnormally expressed, the downstream pathway is abnormally activated, thus promoting the proliferation, differentiation, invasion, and other abnormal activities of tumor cells. The positive rate of abnormal expression of *HER-2* in gastric cancer is 7.3% - 20.2% (14). Moreover, abnormal expression of *HER-2* gene is more common in IGC than in diffuse-type of gastric cancer (DGC) (15). Trastuzumab is a human IgG1 monoclonal antibody from recombinant DNA that selectively binds to the extracellular domain IV of *HER-2*, blocks the abnormal activation of *HER-2*, and then inhibits the downstream signaling pathway and tumor cell proliferation (14). The TOGA trial confirmed that chemotherapy combined with trastuzumab effectively prolonged the median overall survival time of patients with advanced gastric cancer with positive *HER-2* (16).

The efficacy of trastuzumab in HER-2 positive patients has been fully confirmed; however, most of these studies are based on abnormal changes in the primary *HER-2* gene. In this case, abnormal amplification of the *MET* gene was initially observed, but with no abnormality in the *HER-2* gene. After continuous *MET*-targeted therapy, symptoms and examination results showed good curative effect. In later stages, the disease progressed, and new gene abnormalities were found, including *HER-2, TP53*, and *KRAS*. However, the cause of *HER-2* mutations remained unexplored. Similar studies have reported the efficacy of trastuzumab combined with crizotinib in the treatment of *HER-2*-amplified and *MET*-amplified metastatic gastric cancers (17). However, unlike our case, the patient was diagnosed with multiple metastases of gastric adenocarcinoma, and *HER-2* gene amplification was detected. After trastuzumab treatment, *MET* amplification was detected using delayed second-generation sequencing. After combining the two targeted drugs, the symptoms were temporarily relieved (performance status improved from 4 to 2). Another case report suggested that amplification of *MET* gene might be the driving factor for gastric cancer (4). After two months of crizotinib administration in patients with liver metastasis from advanced gastric cancer, the metastasis was significantly controlled and progression free survival (PFS) was prolonged to 20 months.

We believe that *HER-2* mutation in this case was a secondary event. Therefore, the patient responded initially to administration of trastuzumab, but the improvement was not sustained. This might be due to the overactivation of primary *MET*, leading to heterodimer phosphorylation and formation of heterodimers with *EGFR*, *HER-2*, *HER-3*, and *RET* receptor tyrosine kinases, affecting the normal transduction of other signaling pathways (18). The signal triggered by this heterodimer increased the resistance to *EGFR* or *HER-2* targeted therapy. In addition, mutations in *TP53, KRAS, BRAF, RET*, and other genes appeared in the later stages of the disease. The mutated *TP53* gene can enhance *MET* signaling, expression of c-MET protein, and activation of the downstream signaling pathway of *MET* (19). Studies have shown that in HER-2-positive metastatic gastric cancer patients, the *KRAS* mutation rate in drug-resistant patients is also higher than that in sensitive patients, and the activated *KRAS* gene can promote epithelial-mesenchymal cell transformation, promoting the transformation of gastric cancer cells into tumor stem cells, resulting in treatment resistance (20). The patient described in this paper had advanced gastric cancer, and new metastatic lesions continued to appear as the disease progressed. At present, it is considered that for this patient, *MET* gene is the driving gene for gastric cancer. However, because of the spatial and temporal heterogeneity of gastric cancer, it is impossible to determine the heterogeneity of the driving genes among the metastatic foci. With the occurrence of late gene overexpression and mutations, genes interact with each other, including the promoting effect of *KRAS* mutations on the abnormal expression of *MET* and abnormal activation of downstream signaling pathways. The abnormal transformation of the *KRAS* gene in gastric cancer cells, the enhancement of abnormal *MET* signal by *TP53* mutation, and other changes, can create complex interactions. This leads to the unsatisfactory efficacy of gastric cancer for c-met amplification therapy compared with lung cancer.

Owing to the high heterogeneity of gastric cancer, accurate molecular classification is of great significance for its diagnosis, treatment, and prognosis. The realization of accurate molecular therapy for gastric cancer is a direction for future research, but there is still a long way to go.

## Data availability statement

The original contributions presented in the study are included in the article/supplementary material. Further inquiries can be directed to the corresponding authors.

## Ethics statement

Written informed consent was obtained from the individual(s) for the publication of any potentially identifiable images or data included in this article.

## Author contributions

XZ: Writing – original draft, Writing – review & editing, Data curation. XRZ: Writing – original draft, Writing – review & editing, Data curation. DG: Writing – review & editing. CZ: Writing – review & editing. YW: Writing – review & editing. YF: Writing – review & editing. SG: Writing – review & editing. JW: Funding acquisition, Supervision, Writing – review & editing. FZ: Funding acquisition, Supervision, Writing – review & editing.
